# Pain Control in Oral Mucositis According to the Severity Scale: A Narrative Literature Review

**DOI:** 10.3390/jcm14134478

**Published:** 2025-06-24

**Authors:** Nawal Suhaimi, Noor Azura Hani Abdul Razak, Roszalina Ramli

**Affiliations:** 1 Department of Oral and Maxillofacial Surgery, Faculty of Dentistry, Universiti Kebangsaan Malaysia, Kuala Lumpur 50300, Malaysia; nawalsuhaimi@ukm.edu.my; 2 Department of Oral and Maxillofacial Surgery, Hospital Umum Sarawak, Kuching 93586, Malaysia; noorazurahani@yahoo.co.uk

**Keywords:** pain, oral mucositis, stomatitis, pain measurement

## Abstract

Background: Oral mucositis (OM) is a painful and debilitating stomatitis that often arises following head and neck radiotherapy, chemotherapy, or stem cell transplant, leading to treatment delays and potential patient intervention interruption. The aim of this narrative review was to explore the modalities in the management of OM. When supported by evidence, treatment is customized according to its severity. Method: A literature search was performed using the PubMed database. The search strategy consists of keywords such as “pain management”, “pain control”, and “oral mucositis”. Literature references from 1997 to 2024 were selected by the authors based on relevance to the current practice of oral mucositis pain management. Results: Fourteen studies were included in this review. Interventions were classified into pharmacological and non-pharmacological modalities. In relation to pain measurement, for grade 1 OM, topical treatments are the primary recommendation. For grades 2 to 4, where pain is moderate to severe, systemic analgesia should be administered. Honey and oral care are beneficial for OM with grades 2 to 4. Conclusions: Effective management of OM should be tailored to the severity of the condition, incorporating both pharmacological and non-pharmacological strategies. While various modalities have shown promise in relieving symptoms and enhancing the quality of life, a multifaceted, patient-centered approach remains essential. Advancing high-quality clinical trials, particularly those evaluating non-pharmacological interventions will be crucial to expanding treatment options. Future efforts should focus on personalized therapies, integration of combination treatments, adoption of standardized pain assessment tools, and long-term outcome studies to improve clinical effectiveness and optimize patient care.

## 1. Introduction

Oral mucositis (OM) is a debilitating consequence of head and neck radiotherapy, chemotherapy, and hematopoietic stem cell transplant (HSCT). It causes treatment delays and interruption of intervention for the patient. Managing OM is crucial as it can progress to an acute, life-threatening stage secondary to malnutrition, dehydration, and loss of mucosal barrier.

OM affects around 40% of patients undergoing standard-dose chemotherapy, 80% of those receiving radiation therapy to the head and neck, and nearly all patients undergoing HSCT [1]. OM can develop two weeks after radiation initiation, preceded by ulcerations and pseudomembrane formation [2]. Pain generally increases during treatment, which may correlate with worsening mucositis [3]. The pathogenesis involves direct cytotoxic effects of cancer treatments, such as radiation therapy and certain chemotherapeutic agents, which can lead to the destruction of the rapidly dividing cells in the oral mucosa, resulting in the development of painful ulcers and inflammation [4,5]. This process is further exacerbated by the weakening of the mucosal barrier, which leaves the underlying tissues more susceptible to damage and infection [6]. Radiation-induced OM is traditionally understood as an ‘outside–in’ process, where the DNA strand breaks occur in the basal epithelial cells of the oral mucosa [7]. Electron micrographs revealed damage to the endothelial walls within one week following acute radiation in animal models, occurring at least five days prior to epithelial breakdown [8]. In contrast, chemotherapy-induced OM is primarily attributed to basal cell damage caused by the penetration of the drugs into these cells via the submucosal blood supply [7]. Several antineoplastic drugs are known to cause endothelial toxicity, including those with stomatotoxic potential, such as 5-fluorouracil, cisplatin, and melphalan [9,10]. These regimens are used in HSCT, which involves an intense period, unlike radiation therapy, which is delivered in multiple small daily doses [11]. High doses of melphalan, busulfan, and/or cyclophosphamide, with or without total body irradiation, are associated with moderate to severe OM [12,13]. The risk of severe OM may be lower in patients who do not receive total body irradiation [14,15].

### 1.1. Phases of Pathogenesis of Oral Mucositis

**Figure 1:**
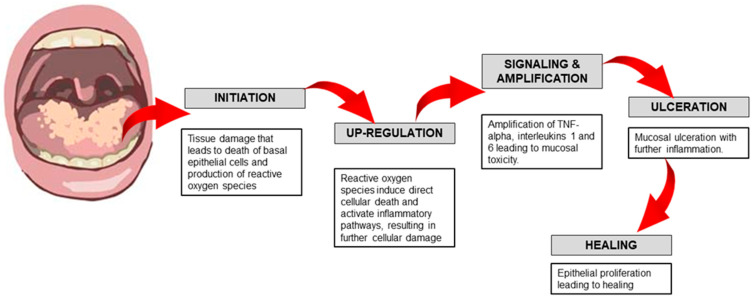
Pathogenesis of mucositis from initiation until healing phase.

The biological complexities of the pathogenesis are simplified into five phases, namely, initiation, upregulation, signaling and amplification, ulceration, and healing, as shown in Figure 1 below.

Initiation of Tissue Injury: Radiation and chemotherapy induce cellular damage, leading to the death of basal epithelial cells. The production of reactive oxygen species (free radicals) by these treatments is also thought to contribute to the onset of mucosal injury [7,11].

Upregulation of Inflammation through Messenger Signals: In addition to directly causing cell death, free radicals activate second messengers that relay signals from cell surface receptors to the cell’s interior. This process enhances the expression of pro-inflammatory cytokines, resulting in tissue damage and further cell death [7,11].

Signaling and Amplification: The increased production of pro-inflammatory cytokines, such as tumor necrosis factor-alpha (TNF-α), primarily by macrophages, contributes to mucosal cell damage. Additionally, this process triggers molecular pathways that further intensify mucosal injury [7,11].

Ulceration and Inflammation: Mucosal ulcerations are accompanied by a significant infiltration of inflammatory cells, partly due to metabolic by-products from the colonizing oral microflora. This secondary infection further elevates the production of pro-inflammatory cytokines [7,11,16].

Healing: This phase involves epithelial proliferation, along with cellular and tissue differentiation, ultimately restoring the integrity of the epithelium [7,11].

### 1.2. Severity of OM

Mucositis is diagnosed primarily through clinical assessment. Two grading scales are commonly used to assess the severity of mucositis: the World Health Organization (WHO) Oral Toxicity Scale [17] and the Radiation Therapy Oncology Group’s Acute Radiation Morbidity Scoring Criteria for Mucosa [18]. The criteria of severity for the two scales are shown in Table 1.

Consequences of OM include pain, erythema, and aspiration [1,2]. This morbid pain may lead to poor oral intake and result in having to use nasogastric or percutaneous endoscopic gastrostomy feeding [1,2]. Moderate to severe OM has been linked to systemic infections and transplant-related mortality [1,2]. In patients with hematologic malignancies undergoing allogeneic HSCT, greater OM severity correlates with a longer duration of total parenteral nutrition and parenteral narcotic use, more days with fever, a higher risk of significant infections, prolonged hospitalization, and increased overall inpatient costs [1].

OM pain also leads to breakthrough pain, which requires additional treatment time, and this leads to actual treatment interruption. The mainstay management of mucositis is symptomatic treatment. While there is no single universally effective treatment, there are definitely evidence-based recommendations available for specific interventions. Oftentimes, when non-pharmacological methods fail to provide significant relief, systemic analgesics are essential [19].

The primary objective of this descriptive review was to explore the management of OM with various therapeutic agents. Additionally, it sought to identify the appropriate agents based on the severity of OM.

## 2. Methods

The aim of this narrative review is to explore the modalities in the management of OM according to its severity. A literature search was conducted using the PubMed database, covering publications from 1997 to 2024. The older literature was included to enhance the comprehensiveness of findings, particularly in relation to severity-based pain control, for which recent data remain limited.

Studies were included if they involved patients of any age with oral mucositis, assessed pain control interventions, and used a recognized scale (e.g., WHO, Radiation Therapy Oncology Group’s Acute Radiation Morbidity Scoring Criteria for Mucosa, or others) to grade the severity of mucositis. Keywords used were “pain management”, “pain control”, and “oral mucositis”. Eligible studies must report pain-related outcomes stratified by severity. These may include randomized controlled trials (RCTs), clinical trials, cohorts, case-control studies, or case series published in English. Conversely, we excluded systematic reviews, editorials, commentaries, letters to editors, abstracts, and non-English texts.

All citations were imported into the Mendeley Reference Manager. Duplicates were identified and removed. The authors screened the publication year, title, research design, keywords, and abstracts, with full-text articles reviewed for details. Disagreements were resolved through consensus.

## 3. Results

A total of 62 articles were identified from the initial search. After screening the titles and abstracts for relevance and conducting a full-text review to assess eligibility, 14 articles were ultimately included. The studies selected for this review are summarized in Table 2. Eight studies involved randomized controlled trials, two clinical trials, two retrospective record studies, and one prospective case series.

The management of OM was divided into pharmacological and non-pharmacological interventions.

### 3.1. Pharmacological Interventions

Analgesic support is fundamental, and opioid therapy remains the mainstay for OM-related pain management during chemotherapy and radiation therapy [20,21].

There are two main branches of pharmacological therapy in managing this pain, namely, topical and systemic agents [19].

#### 3.1.1. Topical Agents

Numerous topical agents have been explored for this cause. These include anesthetic agents, antimicrobial mouthwashes, and hydrating gels [19,22,23,24].

##### Topical Anesthetic Agents

Topical anesthetics are among the most commonly used treatments to alleviate pain associated with OM. These agents work by reducing pain through the local anesthesia effect [19].

- Lidocaine

Lidocaine is available in oral gel or viscous solutions. It can provide prolonged pain relief by blocking sodium channels, inhibiting nerve impulse transmission, and effectively numbing the affected area [22]. Lidocaine is often used in patients undergoing cancer treatments who require significant pain management [22].

One of the main concerns with lidocaine is its potential toxicity if swallowed in large quantities, especially in pediatric patients or those with liver dysfunction. Therefore, careful administration is required, particularly when used in conjunction with other medications.

Lidocaine is commonly included as an ingredient in magic mouthwash. Other components often found in magic mouthwash include diphenhydramine, corticosteroids, magnesium hydroxide/aluminum hydroxide, and nystatin [23]. The formulation of magic mouthwash varies across hospitals, with many institutions developing their specific combinations [23].

Lidocaine can also be incorporated into buccal films together with diclofenac potassium. Lidocaine has an effect of pain reduction in patients. Films are also acceptable to patients, considering they are flexible and comfortable [24];

b. Ketamine

Oral ketamine mouthwash was shown to cause a reduction in pain scores for stomatodynia and odynophagia and significantly improved sleep quality [25];

c. Benzydamine mouthwash

Benzydamine mouthwash works through its anti-inflammatory, anesthetic, and analgesic properties. It has been shown to reduce the production of tumor necrosis factor alpha (TNF-α), interleukin-1 beta (IL-1β), and prostaglandins, as well as act as an antioxidant [26]. It has also been studied in multiple prospective and controlled clinical trials, which reported significantly positive outcomes in head and neck cancer patients undergoing stomatotoxic treatments.

Patients using benzydamine experienced a decrease in the severity of oropharyngeal mucositis compared to those using chlorhexidine [27]. Additionally, benzydamine delayed the onset of higher-grade mucositis compared to chlorhexidine, confirming that benzydamine is most effective when used as a preventive modality [27]. Benzydamine is suitable for Grade 1 mucositis [28].

##### Topical Corticosteroids

Corticosteroids are widely used for their potent anti-inflammatory effects. In the context of OM, topical corticosteroids help reduce the inflammation and swelling that can exacerbate pain. Common corticosteroid includes clobetasole propionate, which is available in the form of an ointment that can be applied directly to the ulcerated mucosa [29]. This agent helps reduce local inflammation and prevents further ulceration, thus helping to alleviate pain. In a randomized controlled trial involving patients with rheumatoid arthritis, clobetasol has been effective in the management of methotrexate-induced oral ulcers [29]. While corticosteroids are effective, they should be used cautiously due to their potential side effects, such as delayed wound healing and an increased risk of oral infections, such as candidiasis.

##### Topical Antiseptics and Antimicrobials

Infections may complicate OM and worsen the pain. Topical antiseptics and antimicrobials are used not only to prevent infection but also to reduce inflammation and pain. Chlorhexidine gluconate is a broad-spectrum antimicrobial agent commonly used in oral care to manage mucositis [22]. It works by reducing bacterial load in the oral cavity, which prevents secondary infections and helps maintain a cleaner environment for healing. Though chlorhexidine does not directly alleviate pain, its role in preventing infections can indirectly reduce discomfort. Although chlorhexidine is generally safe, prolonged use may lead to tooth staining and taste alterations, which some patients may find bothersome. However, a guideline advises against the use of chlorhexidine for patients with Grade 1 radiation mucositis [30].

##### Topical Coatings and Protective Agents

Topical agents that form protective coatings over mucosal lesions can help shield them from mechanical irritation and promote healing. These agents are often used in conjunction with other treatments to provide pain relief by creating a barrier that reduces exposure to irritants such as food and drink [19].

- Hyaluronate gel

Gel-based formulations, such as those containing hyaluronate, have shown merits in reducing the pain or disruption to the oral mucosa [22], commonly used for their protective and soothing effects. In terms of chemotherapy-induced OM, administration of the chemotherapy agent triggered a diffuse erythema and/or ulceration on the oral mucosa. Hyaluronic acid significantly limited OM and reduced the ulcer size, resulting in improvement in the OM [31].

b. Sucralfate

Sucralfate is another agent used to form a protective coating over the ulcerated mucosa [6,32]. It binds to the protein components of the mucosal lining, forming a gel-like barrier that promotes healing and reduces pain. Although it is more commonly used in the gastrointestinal tract, sucralfate has also been employed in OM to reduce irritation and provide pain relief. Similar to chlorhexidine, sucralfate is not recommended for patients with Grade 1 radiation mucositis [28]. The Mucositis Study Group of the Multinational Association of Supportive Care in Cancer and the International Society for Oral Oncology (MASCC/ISOO) guidelines also recommend against the use of sucralfate in radiation-induced OM due to a lack of efficacy [33].

##### Antidepressant Topical Rinse

Doxepin rinses, a tricyclic antidepressant, has shown promising results in Phase 3 randomized controlled trials by significantly reducing OM pain during the first 4 h after administration [34].

#### 3.1.2. Systemic Agents

Systemic agents in this context include opioids, corticosteroids, cytoprotective agents, and growth factors [12,35,36,37,38,39,40,41].

##### Opioids

Opioid analgesics such as morphine, hydromorphine, fentanyl, and oxycodone are the most commonly used drugs for OM. These opioids exert their analgesic effects by binding to specific opioid receptors in the central and peripheral nervous systems. These receptors, known as mu, delta, and kappa opioid receptors, are found throughout the body, including in the oral mucosa. When opioids bind to these receptors, they disrupt the transmission of pain signals, effectively reducing the perception of pain experienced by the patient [35].

With regards to OM, the mechanism of action of systemic opioids is multifaceted. First, opioids alleviate direct pain caused by inflammation and ulceration of the oral mucosa. By dampening the nociceptive (pain-sensing) signals from the affected area, opioids can provide significant relief to the patient [36].

Additionally, opioids may also have indirect effects on OM. Opioids have been shown to have anti-inflammatory properties, which can help reduce the severity of mucosal damage and promote healing [36]. Opioids help improve the patient’s overall comfort and well-being, which can indirectly enhance the healing process and reduce the risk of complications, such as infection [36].

##### Corticosteroids

Saito et al. (2022) showed that dexamethasone significantly reduced the occurrence of all-grade OM in a dose-dependent manner [37]. It was also noted that extended use of dexamethasone reduces taxane-induced acute pain syndrome, primarily driven by inflammation, due to its potent anti-inflammatory properties [37].

##### Cytoprotective Agent

Cytoprotective agents, such as amifostine, have been investigated for their potential role in mitigating the effects of radiation and chemotherapy on oral mucosa [38]. The mechanism of action for amifostine involves reducing reactive oxygen species, which are involved in the development of OM. However, due to issues like methodological flaws or small sample sizes, studies have not consistently shown a reduction in the duration or severity of chemotherapy-induced OM [12].

##### Growth Factor

Granulocyte-macrophage colony-stimulating factor (GM-CSF) is a potent growth factor for the myeloid lineage of hematopoietic cells [39]. It binds to cell membrane receptors, promoting cellular growth, proliferation, and differentiation [39]. However, a recent study found no significant differences in the occurrence or severity of OM between patients who used GM-CSF and those who used a salt and soda regimen [40].

Palifermin is a recombinant human keratinocyte growth factor (KGF) produced using recombinant DNA technology in *Escherichia coli* [12]. It is primarily used in patients with hematological malignancies undergoing myeloablative therapy, a treatment that often causes severe mucositis [12]. Palifermin exerts its effects by stimulating epithelial cell growth and thickening the non-keratinized layers of the oral and gastrointestinal mucosa, thereby reducing the occurrence, duration, and severity of mucositis [12].

The most common side effects of palifermin primarily affect the skin and oral mucosa. These include oral mucosal and tongue papillae enlargement, dysgeusia, paresthesia, mucosal discoloration, rash, itching, erythema, and skin hyperpigmentation [41].

### 3.2. Non-Pharmacological Intervention

#### 3.2.1. Oral Cryotherapy

One of the non-pharmacological approaches is the use of oral cryotherapy, which involves the application of ice chips or ice pops to the oral cavity during chemotherapy infusion. This technique has been shown to reduce the incidence and severity of OM by constricting blood vessels and reducing the delivery of chemotherapeutic agents to the oral mucosa [14]. Ice chips are applied to the mouth 5 min before chemotherapy administration, which continues every 30 min during the course of chemotherapy [42]. Ice popsicles can also be considered apart from ice chips [42].

#### 3.2.2. Low-Level Laser Therapy/Photobiomodulation

Similarly, the use of low-level laser therapy (LLLT) has been explored as a means of promoting wound healing and reducing inflammation in the oral cavity. A study showed that laser application can reduce the occurrence and intensity of OM [43]. This method requires specific equipment and trained individuals. Studies found that 660 nM wavelength, with a 40 mW power output and an energy density of 4 J/cm^2^ irradiation, was beneficial in preventing OM. However, more research is needed for a more defined wavelength and intensity to obtain the best tissue response for this treatment [43].

#### 3.2.3. Oral Capsaicin

A pilot study demonstrated that oral capsaicin taffy provided substantial pain reduction in 11 patients with OM pain [44]. This effect is based on the theory of desensitization. If oral pain is transmitted by the same neurons responsible for capsaicin-induced pain, then desensitizing these neurons with a capsaicin concentration that induces a burning sensation similar to oral pain could potentially alleviate discomfort [44]. Although the pain relief is temporary, optimizing this approach by adjusting the capsaicin concentration may enhance its benefits for patients [44].

#### 3.2.4. Herbal and Natural Remedies

In addition to conventional pharmaceutical agents, various herbal and natural products have been explored for the treatment of OM. These agents may have soothing, anti-inflammatory, or healing properties. Some examples include the following:

- Aloe vera

Aloe vera gel is often used in the treatment of OM due to its well-known anti-inflammatory and wound-healing properties. It is applied topically to the affected areas to reduce pain and accelerate healing. In a study by Puataweeponga et al. (2009), aloe vera juice was given to patients who received conventional radiation therapy, and the mucosal reaction was assessed during that radiation course [45]. Aloe vera juice was shown to be beneficial in alleviating the severity of radiation-induced mucositis without side effects [45].

b. Honey

Honey is another natural product that has demonstrated anti-inflammatory, antimicrobial, and wound-healing properties. Honey can help soothe the painful lesions of OM, reduce inflammation, and prevent infection. Honey is shown to accelerate recovery and healing compared to control treatments in patients with radiation-induced mucositis [46,47]. Additionally, studies showed that honey notably decreased the severity of radiation-induced grade 3–4 mucositis [48,49,50]. A total of 65% of patients using propolis achieved complete healing by day [49], while 98% of those treated with royal jelly were fully healed within 3–4 days [50].

Both honey and propolis exhibit anti-inflammatory and antioxidant properties, inhibit prostaglandin synthesis in mucosal tissue, enhance the immune response by stimulating phagocytic activity and cellular immunity, and promote epithelial tissue healing [49,50]. Moreover, propolis is a rich source of iron and zinc, essential elements for collagen synthesis [49,50]. One study suggested the use of 20 mL of honey, which is swallowed slowly 15 min before and after radiotherapy sessions and later before bed to allow for smearing of honey to oral and pharyngeal surfaces [48]. This protocol was administered from day 1 of radiation till the end of 6 weeks [48].

#### 3.2.5. Comprehensive Oral Care

Another non-pharmacological strategy is the implementation of a comprehensive oral care (COC) protocol, which includes regular assessments and oral hygiene instructions. These protocols aim to maintain oral mucosal integrity, prevent infections, and alleviate symptoms such as pain and dry mouth [51]. COC can be performed thoroughly yet gently for patients with grade 1 OM. However, for more advanced grades, increased pain necessitates a modified approach, limiting COC to gentle oral care. This includes rinsing with saline and using a very soft toothbrush, soft sponge, or cloth for cleaning the teeth, particularly when the pain is severe.

Although these non-pharmacological interventions yield varying outcomes, they offer a holistic and patient-centered approach to OM management. Incorporating these strategies into comprehensive care plans can enhance patients’ quality of life during cancer treatment and alleviate the burden of this debilitating condition [51].

**Table 2:** List of Studies Included in the Review.

No.	Authors and Year	Type of Study	Number of Subjects	Brief Description of Study	Severity of OM	Grading Used	Interventions
1	Alfieri et al., 2016 [21]	Retrospective record study	75	Opioid therapy for patients with oropharyngeal cancer treated with chemoradiotherapy	2 and above	WHO	Weak and strong opioids
2	Shillingburg et al., 2017 [25]	Open-label, prospective, phase II interventional study	30	Patient to gargle with ketamine mouthwash	3–4	WHO	Oral ketamine mouthwash 20 mg/5 mL four times daily and every 4 h
3	Cheng, 2006 [27]	RCT	14	Chlorhexidine vs. Benzydamine mouthwash from 1–14 days completion of RT	2 and above	WHO	Chlorhexidine 0.2% vs. Benzydamine 0.15% mouthwash
4	Ahmed et al., 2019 [29]	RCT	30	Rheumatoid arthritis pts on methotrexate treatment randomly assigned to receive human platelet lysate vs. Clobetasol Propionate	3	WHO	Intervention: human platelet lysate Control: Clobetasol Propionate
5	Sio et al., 2020 [34]	RCT	275	92 patients: doxepin mouthwash (25 mg/5 mL water); 91 patients: diphenhydramine-lidocaine-antacid; 92 patients: placebo.	4 and greater	Pain score (scale, 0–10)	doxepin mouthwash or diphenhydramine-lidocaine-antacid mouthwash
6	Saito et al.; 2022 [37]	Retrospective analysis of medical record	131	Case: High dose of dexamethasone (9.9 mg infusion on day 1 and 8 mg orally on days 2–4) Control: Low dose of dexamethasone (6.6 mg infusion on day 1 and 4 mg orally on days 2–4	1–2	WHO	Dexamethasone infusion
7	Dodd et al., 2022 [40]	RCT	91	1. GG (GM CSF/GM CSF): GM CSF mouthwash during both prevention and treatment phases. 2. SS (Salt and Soda/Salt and Soda): Salt and soda mouthwash throughout. 3. SG (Salt and Soda then GM CSF): Salt and soda during prevention, switching to GM CSF at the onset of mucositis	unspecified	Radiation Therapy Oncology Group’s Acute Radiation Morbidity Scoring Criteria	Granulocyte-macrophage colony-stimulating factor (GM-CSF) mouthwash
8	Vadhan-Raj et al., 2010 [41]	RCT	48	Case: Palifermin as a single dose before each cycle of chemotherapy Control: Placebo	2–4	WHO	Palifermin (180 µg per kg of body weight) administered intravenously as a single dose 3 days before each chemotherapy cycle (maximum, 6 cycles)
9	Silva et al., 2011 [43]	RCT	42	Case: Low light laser therapy and oral hygiene protocol Control: Oral hygiene protocol	0–4	WHO	Low-level laser therapy 600 nm irradiation
10	Berger et al., 1995 [44]	Prospective case series	11	Patients were instructed to allow the candy to dissolve in the mouth	1–4	Easter Cooperative Oncology Group	Capsaicin taffy
11	Puataweeponga et al., 2009 [45]	Phase II trial	61	Case: Aloe vera juice Control: Placebo	0–4	Radiation Therapy Oncology Group’s Acute Radiation Morbidity Scoring Criteria	Aloe vera juice
12	Amanat et al., 2017 [48]	RCT	82	Case: Honey Control: Saline	0–4	Radiation Therapy Oncology Group’s Acute Radiation Morbidity Scoring Criteria	20 mL of honey daily during radiotherapy
13	AkhavanKarbassi et al., 2016 [49]	RCT	40	Case: Propolis mouthwash Control: Placebo	0–4	WHO	30% Propolis extract mouthwash (5 mL swished for 60 s and expectorated three times daily for 7 days)
14	Erdem & Güngörmüş, 2014 [50]	RCT	103	Case: Royal jelly with mouthwash therapy (Benzydamine hydrochloride and nystatin rinse) Control: Mouthwash therapy	1–3	WHO	Royal jelly, swished for 30 s and swallowed, daily dose of 1 g, twice a day

Abbreviations: RCT = randomized controlled trial; WHO—World Health Organization.

Table 3 below summarizes the agents discussed, highlighting their suitability according to the WHO oral mucositis grading. Agents that were mentioned without a corresponding severity grade are not included in this figure. For grade 1 OM, most recommended treatments are in the topical form. In grades 2 to 3 OM where pain is typically moderate to severe, systemic analgesia is indicated according to the WHO pain ladder. For grade 4 OM, treatment usually involves systemic opioids. The only documented non-pharmacological modalities for grades 2 to 4 are honey and oral care.

## 4. Discussion

The severity of OM varies widely among patients, with some individuals demonstrating greater tolerance to the associated pain and discomfort [52]. Several factors contribute to this variation in pain tolerance. Patient-specific characteristics, including tumor type, age, oral health, nutritional status, and organ function, all influence the severity of symptoms [12]. Additionally, the choice of chemotherapeutic agents plays a significant role, as certain drugs, such as antimetabolites and purine analogs, are more strongly linked to a higher occurrence of OM [12]. The use of combination chemotherapy regimens can further aggravate the condition, resulting in heightened pain for affected patients [12].

Beyond clinical factors, the subjective experience of pain is also shaped by the psychological and social determinants of the patients. Patients’ perceptions of pain, coping mechanisms, and access to supportive care all contribute to the variability observed in pain tolerance.

Patient-reported outcome data play a crucial role in the comprehensive assessment of OM severity. The 0 to 10 Pain Severity Scale is a widely used assessment tool due to its simplicity and ease of administration. The patient is encouraged to report pain severity daily, allowing for the treatment to be adjusted accordingly.

Table 3 provides an overview of the recommended interventions for each WHO oral mucositis grade. However, many interventions lack specific guidance on their suitability based on these grading criteria.

A systematic review conducted by the Multinational Association of Supportive Care in Cancer (MASCC) and the International Society of Oral Oncology (ISOO) in 2019 highlighted the effectiveness of benzydamine 0.15% mouthwash in preventing oral mucositis (OM) in patients receiving up to 50 Gy of radiotherapy or chemotherapy [53]. Benzydamine 0.15% is recommended for the management of oral mucositis (OM), whereas chlorhexidine mouthwash is contraindicated. This is not only due to its lack of efficacy [30] but also because chlorhexidine has been linked to an earlier onset of OM compared to sodium bicarbonate solution [54]. Similarly, sucralfate is generally not recommended due to insufficient evidence of effectiveness [28,33]. However, a recent meta-analysis identified topical sucralfate as one of the most effective interventions for the prevention of OM [32], suggesting the need for further investigation.

Additionally, cryotherapy is recommended for patients undergoing autologous hematopoietic stem cell transplantation (HSCT) when administered alongside a bolus injection of high-dose melphalan or fluorouracil (5FU) [28,55]. Various cryotherapy protocols have been reported in the literature, differing in the duration for which ice cubes or iced water should be held in the mouth, yet all have demonstrated efficacy [55].

Among non-pharmacological interventions, options such as honey, herbal compounds, saliva stimulants, probiotics, and other miscellaneous agents have been explored. In 2020, the Mucositis Study Group of MASCC/ISOO recommended the use of honey, both topically and systemically, for preventing OM in head and neck cancer patients undergoing radiotherapy or combined radiotherapy–chemotherapy [56].

For severe pain management, patient-controlled analgesia involves an intravenous morphine infusion with a patient-controlled pump [56]. Additionally, a topical 0.2% morphine rinse has been suggested as an effective option for alleviating pain associated with radiotherapy-induced OM [57,58].

Basic oral care, which encompasses routine oral hygiene practices aimed at preventing infections and ensuring comfort, is recommended for all patients, regardless of the type of treatment or stage of OM [56].

There is a notable lack of evidence supporting the efficacy of multimodal strategies. Emerging therapies targeting specific pathways, such as keratinocyte growth factor analogs and cytokine inhibitors, show promise in reducing inflammation and tissue injury [59]. In parallel, innovative delivery systems like mucoadhesive films and nanotechnology-based carriers are being developed to provide localized and sustained pain relief [60]. The use of probiotics to modulate the oral microbiome may also help preserve mucosal integrity and mitigate injury [61]. Photobiomodulation therapy (PBMT) continues to be refined for wider clinical application, while insights into central sensitization highlight a potential role for neuropathic agents such as gabapentinoids [62]. Moving forward, research should focus on personalized treatment approaches, the integration of combination therapies, the use of standardized pain assessment tools, and long-term outcome studies to enhance both clinical effectiveness and patient quality of life.

### Limitations of This Review

While we aim to provide a comprehensive overview of pharmacological and non-pharmacological agents for managing pain in OM, several limitations must be acknowledged. First, this review may be constrained by the availability and quality of existing studies, with potential biases in article selection and variability in study designs, patient populations, and treatment protocols. Selection bias in this review may be attributed to the review design and the exclusion of non-English publications.

Additionally, the efficacy of certain interventions, particularly from the non-pharmacological options, lacks strong support from high-quality, large-scale clinical trials.

Addressing this unmet need could broaden treatment options and improve patient outcomes beyond pharmacological management.

Furthermore, this review does not fully address the long-term safety and side effects of pharmacological treatments, such as opioids and corticosteroids, which could significantly impact patients’ quality of life. Lastly, disparities in healthcare resources and accessibility across different regions may influence the feasibility of implementing certain treatment strategies.

## 5. Conclusions

The management of oral mucositis should be tailored according to severity, with topical agents being effective for grade 1, and systemic opioids commonly used for grades 2 to 4 due to increasing pain intensity. Supportive measures such as honey and diligent oral care have shown added benefits, particularly in moderate to severe cases. While current strategies largely rely on single-modality treatments, future research should explore multi-modality approaches and personalized therapies. Promising developments include targeted agents, innovative drug delivery systems, probiotics, photobiomodulation therapy, and treatments addressing neuropathic pain components. Standardized pain assessment tools and long-term studies will be essential to improving clinical outcomes and enhancing patients’ quality of life.

## Figures and Tables

**Table 1:** Grading Scales by the World Health Organization and National Cancer Institute.

World Health Organization OM Scale [17]	
Grade	Description
0	No mucositis
1	Soreness/erythema
2	Erythema and ulcers but able to tolerate solid diet.
3	Unable to tolerate solids but able to tolerate liquids
4	Unable to tolerate solids or liquids. Oral alimentation is not possible.
Radiation Therapy Oncology Group’s Acute Radiation Morbidity Scoring Criteria for Mucosa [18]	
0	No change over baseline
1	Injection/may experience mild pain not requiring analgesic
2	Patchy mucositis, which may produce an inflammatory serosanguinitis discharge/may experience moderate pain requiring analgesia
3	Confluent fibrinous mucositis/may include severe pain requiring narcotic
4	Ulceration, hemorrhage, or necrosis

**Table 3:** Summary of the pharmacological and non-pharmacological agents for pain relief according to severity of OM.

WHO Mucositis Grade	Pharmacological Agents	Non-Pharmacological Agents
1	Topical anesthetic agents [28] Topical corticosteroids [28,29,37] Topical antiseptics and antimicrobials [27,28] Topical coatings and protective agents [31]	Comprehensive oral care [28] Cryotherapy [28]
	Not recommended: Sucralfate [28,33] Chlorhexidine [30]	
2–3	Topical anesthetic agents [25,28] Systemic corticosteroids [37] Systemic analgesia based on WHO pain ladder [21,28]	Gentle oral care [28] Honey [48,49,50]
4	Topical anesthetic agents [25] Systemic corticosteroid [37] Systemic analgesia at regular intervals [21]	Gentle oral care [28] Honey [48,49,50]

## Data Availability

The data presented in this study are available on request from the corresponding author.
